# L-β-aminoisobutyric acid (L-BAIBA) in combination with voluntary wheel running exercise enhances musculoskeletal properties in middle-age male mice

**DOI:** 10.18632/aging.206325

**Published:** 2025-10-01

**Authors:** Julian A. Vallejo, Yukiko Kitase, Thiagarajan Ganesh, Mark Dallas, Yixia Xie, David S. Moore, Mark L. Johnson, Lynda F. Bonewald, Michael J. Wacker

**Affiliations:** 1 Department of Biomedical Sciences, School of Medicine, University of Missouri, Kansas, MO 64108, USA; 2 Department of Oral and Craniofacial Sciences, School of Dentistry, University of Missouri, Kansas, MO 64108, USA; 3 Department of Anatomy, Cell Biology and Physiology, School of Medicine, Indiana University, Indianapolis, IN 46202, USA; 4 University of Missouri-Kansas City, Department of Civil and Mechanical Engineering, Kansas, MO 64110, USA; 5 Department of Orthopaedic Surgery, School of Medicine, Indiana University, Indianapolis, IN 46202, USA

**Keywords:** endurance exercise, dietary supplementation, musculoskeletal adaptation, electrocardiogram, aging

## Abstract

Contracting skeletal muscles secrete the metabolite L-β-aminoisobutyric acid (L-BAIBA), which when supplemented in the diet can mitigate disuse-induced musculoskeletal dysfunction. However, the effects of L-BAIBA supplementation alone and combined with exercise on cardiac and musculoskeletal properties are currently unknown. We hypothesized that exercise with L-BAIBA supplementation would promote greater cardiac and musculoskeletal benefits than exercise alone. To investigate this hypothesis, we subjected 12-month-old (as a model of middle-age) male C57BL6 mice to voluntary wheel running (VWR) with L-BAIBA (100mg/kg/day) (VWR+L-BAIBA), VWR alone, L-BAIBA alone, or none (CTRL) for three months. After the intervention, conscious electrocardiogram showed slightly prolonged QTc in VWR+L-BAIBA mice compared to CTRL (*p*<0.05). Soleus muscles from VWR+L-BAIBA, but not VWR, were larger, contracted more forcefully, and contained more slow-oxidative type I myofibers compared to CTRL (*p*<0.05). In EDL muscle, VWR but not VWR+L-BAIBA improved fatigue resistance and caffeine-induced recovery (*p*<0.05). In bone, VWR+L-BAIBA but not VWR showed lower bone marrow adiposity, higher trabecular thickness, and connectivity, smaller bone diameter and Moment of Inertia, but higher Modulus of Elasticity than CTRL (*p*<0.05), suggesting L-BAIBA delays aging-induced periosteal expansion due to better bone material qualities. These findings suggest a physiological interaction between exercise and L-BAIBA supplementation to improve soleus muscle and bone properties and reduce bone marrow adiposity.

## INTRODUCTION

Exercise is important for the maintenance of individual health across aging. The health of the musculoskeletal system, in particular, plays a key part in one’s functional ability and longevity. As such, physical inactivity contributes to more than one-third of annual healthcare expenditures in North America alone, as assessed in a 2013 study, and is projected to contribute to 500 million new cases of preventable noncommunicable diseases by 2030 [1, 2]. The decline of the musculoskeletal system initiates sequelae of local and systemic tissue dysfunction, which can ultimately culminate in disability. Skeletal muscle weakness increases the likelihood of suffering a traumatic fall with skeletal fracture, leading to decreased quality of life and increased morbidity and mortality [3, 4]. The burden of musculoskeletal disorders is staggering, affecting up to 1 in 2 individuals worldwide, and is the leading cause of disability [5]. Musculoskeletal decline begins as early as middle age [6, 7], highlighting the importance of maintaining an adequate functional capacity during this period to promote healthy aging [8]. Regular exercise training is effective in improving aging-related decline in muscle and bone properties [9, 10]. High-impact activities such as resistance exercises are favored to promote the largest anabolic enhancement in bone/muscle mass and strength [11, 12]. Aerobic exercises, on the other hand, are especially effective in improving cardiac health and other aspects of musculoskeletal aging, including metabolic dysfunction [12]. However, implementing exercise with aging can be difficult in those with already compromised musculoskeletal function [13]. Moreover, overall adaptation of musculoskeletal tissue to physical activity is a process that loses its vigor with aging [14, 15].

The musculoskeletal unit plays dual roles in providing the mechanical components for physical exercise and functioning as endocrine organs regulating distant and local tissue physiology. Bone has been found to act in an endocrine manner, with osteocytes and osteoblasts releasing hormone-like factors into the circulation that can influence heart and skeletal muscle development and function, including prostaglandin E2, Wnts, and osteocalcin [16, 17]. In addition, skeletal muscles release signaling molecules in response to exercise that confer systemic health benefits as well as positively regulate bone and heart, including the proteins interleukin-6 [18, 19], interleukin-15 [20], irisin [21], and the metabolite β-aminoisobutyric acid (BAIBA). BAIBA is a non-proteinogenic β-amino acid produced during the metabolism of valine or thymine, and its production is tightly regulated by the activity of peroxisome proliferator-activated receptor gamma coactivator 1-alpha (PGC-1α) in muscle [22]. BAIBA has been shown to increase brown adipose-specific gene expression [22], attenuate insulin resistance and hepatic endoplasmic reticulum stress in type II diabetes mellitus [23], and prevent diet-induced obesity [24], suggesting BAIBA mediates the benefits of exercise and is a potential treatment of metabolic disorders.

BAIBA production results in two distinct isomeric structures, L-BAIBA and D-BAIBA. We have reported that the L-isomer of BAIBA is predominantly secreted from both slow-twitch and fast-twitch murine skeletal muscles in response to contractile activity regardless of sex or age [25]. L-BAIBA has shown several protective effects in the heart and musculoskeletal tissues. Cardio-protective properties of L-BAIBA treatment were reported in a mouse model of myocardial infarction-induced heart failure, including downregulation of markers for cardiomyocyte apoptosis and metabolic stress [26]. Previously, we found that L-BAIBA supplementation protected against the deterioration of structural and functional properties of bone and skeletal muscle during prolonged periods of disuse in mice [25]. Additionally, L-BAIBA promoted protection against oxidative stress-induced bone cell death in osteocytes by signaling through its receptor, mas-related G-protein coupled receptor type D (MRGPRD) [25]. A recent study found that elevating serum L-BAIBA through supplementation for two weeks increased grip strength and sensitized the skeleton to sub-optimal mechanical loading by promoting bone formation and anabolic signaling [27]. Therefore, L-BAIBA may also have synergistic properties when combined with other physiological anabolic processes.

Endocrine and paracrine crosstalk between skeletal muscle and other organs during exercise is important for maintaining homeostasis, and L-BAIBA appears to play a key role in this process. While the protective effects of L-BAIBA have been documented in disease models, the effects of L-BAIBA supplementation when combined with exercise in healthy animals have not yet been explored. Therefore, we hypothesized that the combination of exercise with L-BAIBA treatment would promote greater benefits to musculoskeletal and cardiac tissue than exercise alone. To test our hypothesis, we subjected 12-month-old male mice (as a model of middle-age) to sedentary conditions or voluntary wheel running exercise (VWR) with and without L-BAIBA supplementation (100 mg/kg/day) for three months. After the three-month intervention period, structural and functional properties of the heart, fast and slow-twitch skeletal muscles, and bones were assessed.

## RESULTS

**Figure 1:**
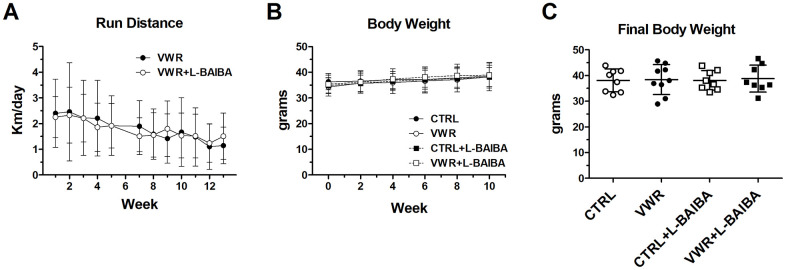
**Run distance and body weight during the three-month intervention period.** (**A**) Average daily running distance of exercising mice after each week over the three-month study. (**B**) Average body weight over the course of the three-month intervention period. Two-way ANOVA, *p*>0.05. (**C**) Average body weight of mice at the end of the three-month intervention period. One-way ANOVA, *p*>0.05. n = 8 CTRL, n = 9 VWR, n = 8 CTRL+L-BAIBA, n = 8 VWR+L-BAIBA. Mean ± SD.

The weekly running distances for the VWR and VWR+L-BAIBA mice are shown in Figure 1A. Weekly running distances were similar between mice performing VWR alone and mice performing VWR with L-BAIBA supplementation. The average running distance was 1.81 km/day and 1.77 km/day, with a peak distance of 2.46 and 2.33 km/day for VWR and VWR+L-BAIBA mice, respectively. The run distance gradually decreased over the study period in both groups, which was reduced to 1.15 and 1.51 km/day during the last week for VWR and VWR+L-BAIBA mice, respectively. Average body weights before the study, during the course of the study, and at the time of sacrifice at 15 months of age showed no differences between any of the groups (Figure 1B, 1C).

### Heart ECG properties and size

Long-term running exercise has been previously found to induce physiological adaptations in the heart including lowered resting heart rate, enhanced heart rate variability (HRV), and cardiac hypertrophy in both humans and rodent models [28–30]. Heart rate and HRV, as assessed by ECG in conscious mice after the three-month intervention were not altered across any of the groups (Supplementary Figure 1A, 1B). Next, cardiac depolarization/repolarization properties, including PR interval, QRS interval, QT interval and corrected QT interval (QTc), were analyzed. PR and QRS intervals were not significantly affected across any of the groups (Supplementary Figure 1C, 1D), indicating no changes to atrioventricular coupling or ventricular depolarization, respectively. Although QT interval was also not different among groups, QTc was significantly prolonged with VWR+L-BAIBA (44.9 ± 1.4 ms) compared to CTRL mice (43.2 ± 1.6 ms, *p*=0.0446), suggesting changes to ventricular repolarization properties (Supplementary Figure 1E, 1F). Lastly, to determine the effect of the VWR exercise and L-BAIBA supplementation in combination and alone on heart size, we measured heart weight and heart weight normalized to body weight (HW/BW) at the end of the three-month intervention. Heart weights and HW/BW were not significantly different with three months of VWR and/or L-BAIBA supplementation (Supplementary Figure 2A, 2B).

### Skeletal muscle size and contractility

**Figure 2:**
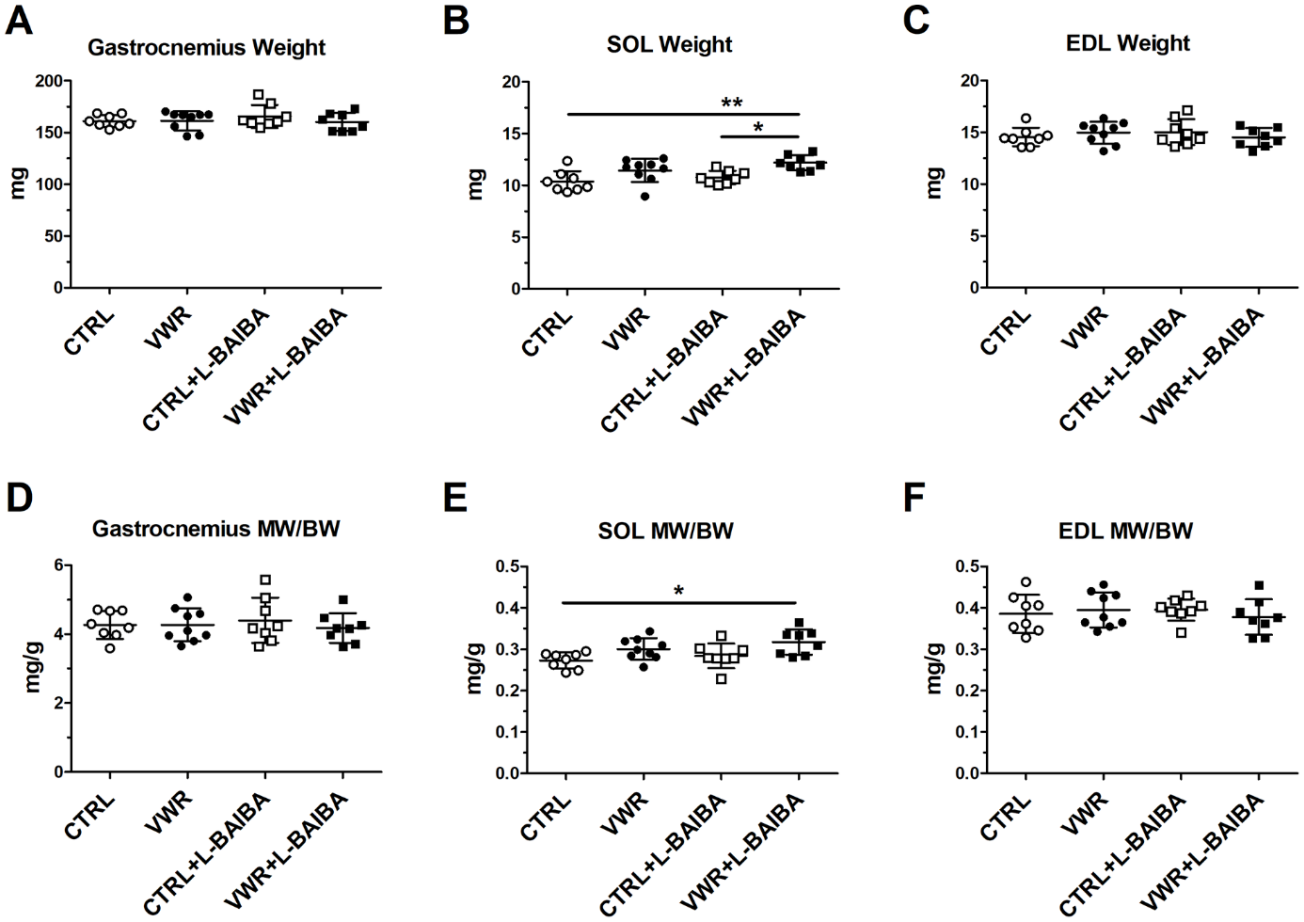
**Muscle weights after the three-month intervention period.** Average muscle weight for gastrocnemius (**A**), soleus (SOL) (**B**), and extensor digitorum longus (EDL) (**C**). Average muscle weight to body weight ratio (MW/BW) for gastrocnemius (**D**), soleus (**E**), and EDL (**F**). **p<0.01, *p<0.05, one-way ANOVA with Tukey. n = 8 CTRL, n = 9 VWR, n = 8 CTRL+L-BAIBA, n = 8 VWR+L-BAIBA. Mean ± SD.

The impact of VWR exercise and L-BAIBA supplementation in combination and alone on skeletal muscle tissue size and contractile function was determined in isolated representative skeletal muscles. First, gastrocnemius muscle, which represents a mixed slow and fast-twitch muscle, did not display any significant changes in absolute weight or muscle weight normalized to body weight (MW/BW) among any of the groups (Figure 2A, 2D). In the slow-twitch soleus muscle, average muscle weight and MW/BW were not statistically significantly changed with VWR alone; however, the VWR+L-BAIBA group displayed increases in soleus weight (12.2 ± 0.7 mg) which were statistically significantly different when compared to sedentary CTRL (10.4 ± 1.0 mg, *p*=0.002) and CTRL+L-BAIBA (10.8 ± 0.6 mg, *p*=0.017) (Figure 2B). Furthermore, soleus MW/BW was significantly elevated with VWR+L-BAIBA (0.32 ± 0.03 mg/g) compared to CTRL (0.27 ± 0.02 mg/g, *p*=0.013) (Figure 2E). There were no differences in fast-twitch EDL muscle weight or MW/BW among any of the groups (Figure 2C, 2F).

**Figure 3:**
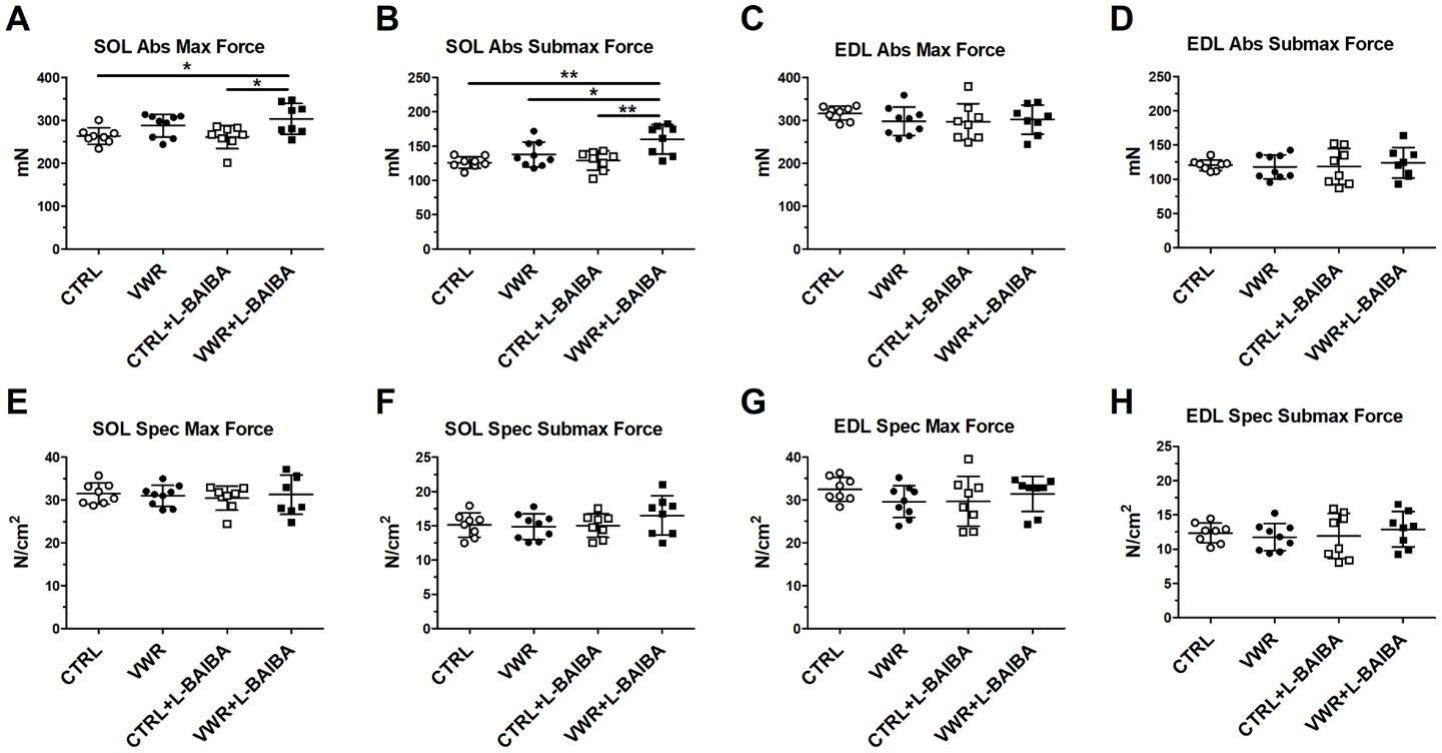
***Ex vivo* muscle contractile force after the three-month intervention period.** Soleus (SOL) absolute maximal contractile force (**A**) and absolute submaximal contractile force (**B**). EDL absolute maximal contractile force (**C**) and absolute submaximal contractile force (**D**). Soleus specific maximal contractile force (**E**) and specific submaximal contractile force (**F**). EDL specific maximal contractile force (**G**) and specific submaximal contractile force (**H**). **p<0.01, *p<0.05, one-way ANOVA with Tukey. n = 8 CTRL, n = 9 VWR, n = 8 CTRL+L-BAIBA, n = 8 VWR+L-BAIBA. Mean ± SD.

**Figure 4:**
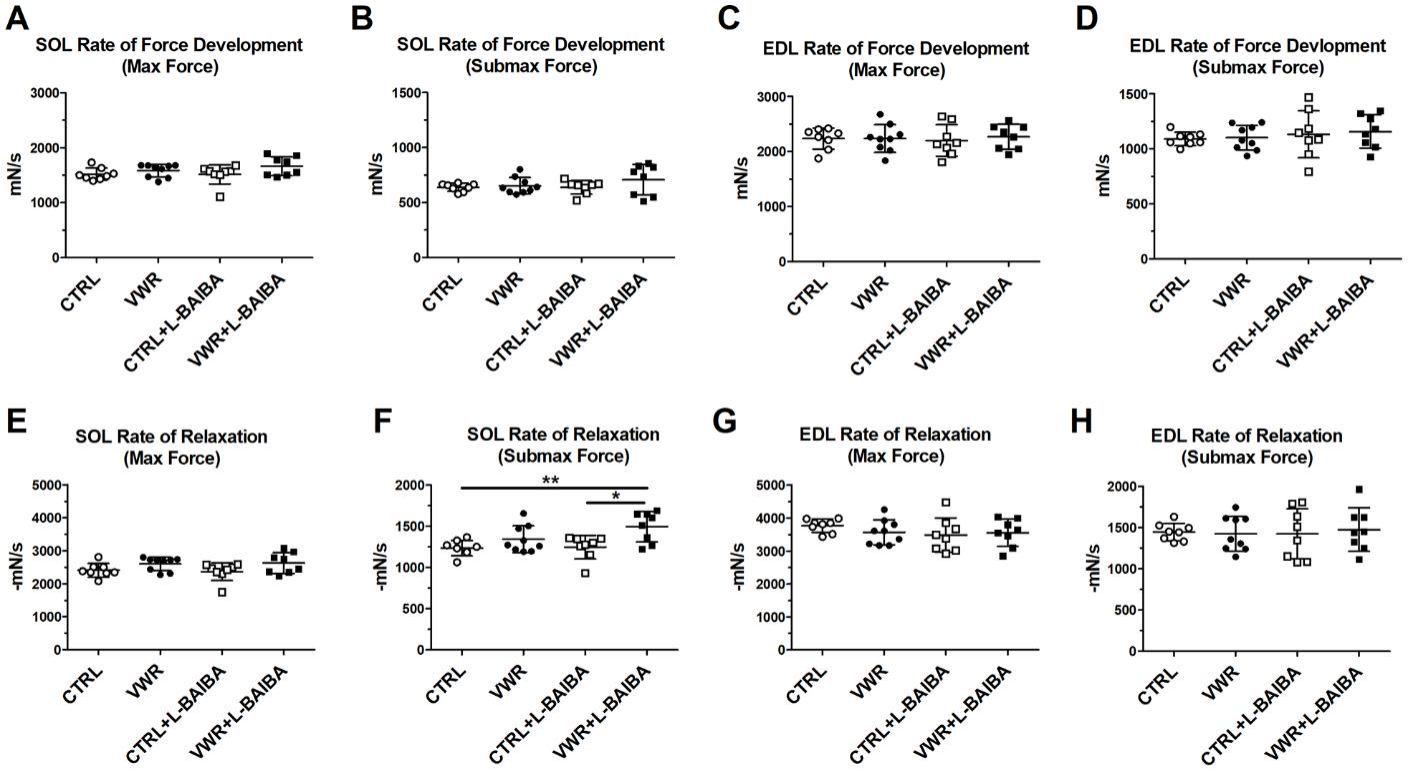
***Ex vivo* muscle kinetic properties of contraction after the three-month intervention period.** Kinetic properties of muscle contraction including the rate of force development for soleus (SOL) maximal force (**A**), soleus submaximal force (**B**), EDL maximal force (**C**), and EDL submaximal force (**D**). The rate of relaxation for soleus maximal force (**E**), soleus submaximal force (**F**), EDL maximal force (**G**), and EDL submaximal force (**H**). **p<0.01, *p<0.05, one-way ANOVA with Tukey. n = 8 CTRL, n = 9 VWR, n = 8 CTRL+L-BAIBA, n = 8 VWR+L-BAIBA. Mean ± SD.

Upon evaluation of *ex vivo* contractility parameters, the VWR group did not display any statistically significant changes to maximal contractile force (VWR: 288.0 ± 26.6mN vs. CTRL: 263.6 ± 19.1mN, *p*=0.2901) (Figure 3A) and submaximal contractile force (VWR: 137.9 ± 18.3mN vs. CTRL: 126.0 ± 8.5mN, *p*=0.4539) (Figure 3B) compared to CTRL mice. However, soleus contractile force was markedly greater in the VWR+L-BAIBA group, which showed statistically significant 15% and 27% increases in absolute maximal and submaximal strength, respectively, compared to both sedentary CTRL and CTRL+L-BAIBA groups (Figure 3A, 3B). Additionally, soleus submaximal contractile force was statistically significantly higher by 16% in the VWR+L-BAIBA group (160.0 ± 21.6mN) compared to VWR alone (*p*=0.0459) (Figure 3B). On the other hand, fast-twitch EDL muscles did not display any significant alterations to absolute contractile force among any of the groups in this study (Figure 3C, 3D). To determine if the significant changes in muscle strength with VWR+L-BAIBA were due to the increased muscle size, the contractile force was normalized to the physiological cross-sectional area of the muscle (specific force). Specific forces were similar among all groups, indicating that the gains in soleus absolute strength with VWR+L-BAIBA were explained by increased abundance of muscle contractile tissue (Figure 3E, 3F). With regard to the kinetic properties of contraction, the soleus muscle rate of force development was not affected by VWR and/or L-BAIBA supplementation (Figure 4A, 4B). The soleus rate of relaxation at submaximal force, on the other hand, was significantly faster by 21% in the VWR+L-BAIBA group, but not with VWR alone, compared to CTRL and CTRL+L-BAIBA groups (Figure 4F). There were no differences in kinetic properties of contraction in EDL muscle (Figure 4C, 4D, 4G, 4H).

Characteristics of muscle excitation-contraction coupling were further evaluated by measuring relative muscle force production across increasing stimulation frequencies (force-frequency). The force-frequency of the soleus muscle was shifted to the left in the VWR+L-BAIBA group at the submaximal stimulatory frequencies of 40 Hz (53.6 ± 5.9%) and 60 Hz (77.2 ± 3.7%) compared to sedentary CTRL (40Hz: 48.0 ± 2.8%, *p*<0.0001; 60Hz: 72.4 ± 1.7%, *p*=0.0009) and at 40 Hz in the VWR+L-BAIBA group compared to VWR alone (48.7 ± 3.1%, *p*=0.0002) (Supplementary Figure 3A). EDL muscles did not show any differences within the force-frequency relationships with exercise and/or L-BAIBA supplementation (Supplementary Figure 3B).

Muscle contractility is dependent upon the release of calcium from internal stores, which can become depleted during repetitive activity. Store-operated calcium entry (SOCE) is important for replenishing internal calcium stores from extracellular calcium in order to maintain muscle contractile function. Muscle contractile force was thus measured in calcium-free buffer followed by the reintroduction of normal calcium levels to determine slow and fast-twitch muscle dependence on external calcium and utilization of SOCE. No significant differences were observed in either soleus or EDL muscle performance during calcium-free conditions or following restoration of normal calcium across any of the different interventions evaluated (Supplementary Figure 4A–4D).

**Figure 5:**
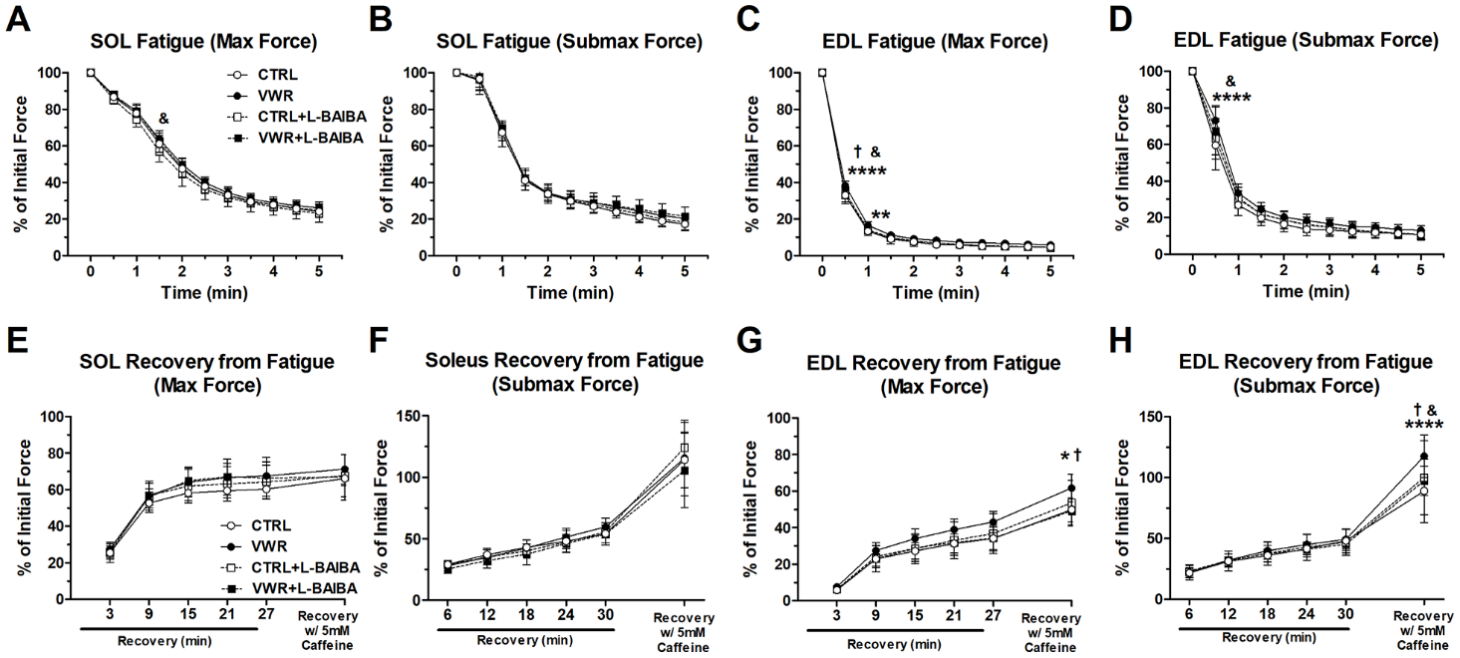
***Ex vivo* muscle fatigue and recovery from fatigue after the three-month intervention period.** Fatigue of contractile force in soleus (SOL) muscle at maximal force (**A**) and submaximal force (**B**), and in EDL muscle at maximal force (**C**) and submaximal force (**D**). ****p<0.0001, **p<0.01 CTRL vs. VWR; †*p*<0.001 VWR vs. VWR+L-BAIBA; &*p*<0.05 VWR vs. CTRL+L-BAIBA. Recovery of contractile force after fatigue in soleus muscle at maximal force (**E**) and submaximal force (**F**), and in EDL muscle at maximal force (**G**) and submaximal force (**H**). Data is expressed as a percentage of initial force at baseline before fatigue. ****p<0.0001, *p<0.05 CTRL vs. VWR; †*p*<0.01 VWR vs. VWR+L-BAIBA; &*p*<0.05 VWR vs. CTRL+L-BAIBA, two-way ANOVA with Bonferroni. n = 8 CTRL, n = 9 VWR, n = 8 CTRL+L-BAIBA, n = 8 VWR+L-BAIBA. Mean ± SD.

Muscle fatigue represents the acute loss of contractile performance experienced with repetitive physical activity, which can be improved with regular endurance exercise. We, therefore, determined the effectiveness of three months of endurance exercise and/or L-BAIBA supplementation on improving muscle fatigue resistance and the ability to recover contractile force following a bout of fatigue. In the soleus muscle, VWR alone significantly improved resistance to acute fatigue of maximal force at the 1.5-minute timepoint (63.6 ± 3.4%) compared to the CTRL+L-BAIBA group only (56.9 ± 5.7%, *p*=0.014) (Figure 5A). There were no effects of VWR and/or L-BAIBA supplementation on the fatigue profile of soleus muscles contracted at submaximal force (Figure 5B). In the EDL muscle, VWR alone resulted in significantly improved resistance to acute fatigue at maximal force during the 0.5-minute timepoint (38.3 ± 2.4%) of the fatiguing protocol compared to CTRL (32.9 ± 4.7%, *p*<0.0001), CTRL+L-BAIBA (33.2 ± 3.7%, *p*<0.0001) and VWR group (34.6 ± 2.8%, *p*=0.0003), and at the one-minute timepoint (16.5 ± 2.1%) compared to CTRL only (13.3 ± 2.2%, *p*=0.0029) (Figure 5C). With regard to EDL submaximal force fatiguability, VWR enhanced resistance to fatigue at the 0.5-minute timepoint (73.2 ± 8.1%) compared to CTRL (59.5 ± 13.3%, *p*<0.0001) and CTRL+BAIBA groups (63.2 ± 11.2%, *p*=0.002) (Figure 5D).

After the muscle fatigue protocol, the magnitude of contractile force recovery was examined over 30 minutes and after the addition of caffeine to the muscles to induce sarcoplasmic reticulum calcium store release and gauge maximal possible force recovery. In soleus, recovery from fatigue and recovery with caffeine treatment at maximal and submaximal contractile forces were not significantly altered by any of the interventions (Figure 5E, 5F). In the EDL muscle, there were no differences during the physiological recovery form fatigue period among groups (Figure 5G, 5H). However, the recovery of EDL muscle maximal force in the presence of caffeine was significantly greater with VWR alone (61.7 ± 7.5%) compared to sedentary CTRL (49.9 ± 6.7%, *p*=0.0119) and VWR+L-BAIBA mice (49.1 ± 7.5%, *p*=0.0047) (Figure 5G). Similarly, EDL muscle submaximal force showed greater recovery with caffeine treatment with VWR alone (117.7 ± 17.5%) compared to CTRL (89.1 ± 26.2%, *p*<0.0001) and VWR+L-BAIBA (97.3 ± 12.0%, *p*=0.0088) (Figure 5H).

### Skeletal muscle histology and fiber type

**Figure 6:**
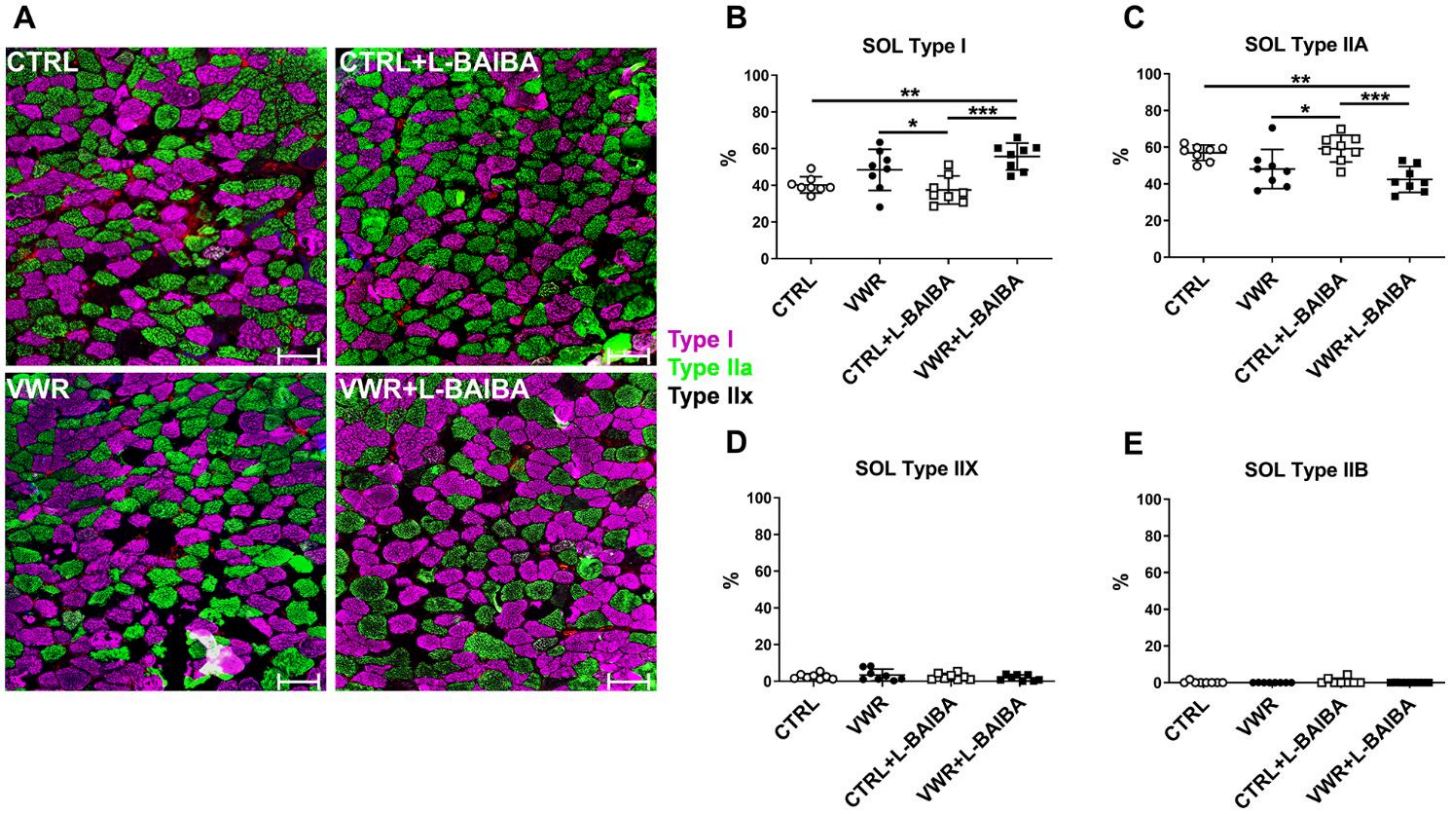
**Soleus muscle fiber type analysis after the three-month intervention period.** (**A**) Representative images of soleus (SOL) muscle histological sections stained for type I (pink), type IIA (green), type IIX (no staining), or type IIB (red) myosin heavy chain. The scale bars represent 100 μm. Quantitation of the percentage of type I (**B**), type IIA (**C**), type IIX (**D**), or type IIB (**E**) myofibers. ***p<0.001, **p<0.01, *p<0.05, one-way ANOVA with Tukey. n = 8 CTRL, n = 8 VWR, n = 8 CTRL+L-BAIBA, n = 8 VWR+L-BAIBA. Mean ± SD.

**Figure 7:**
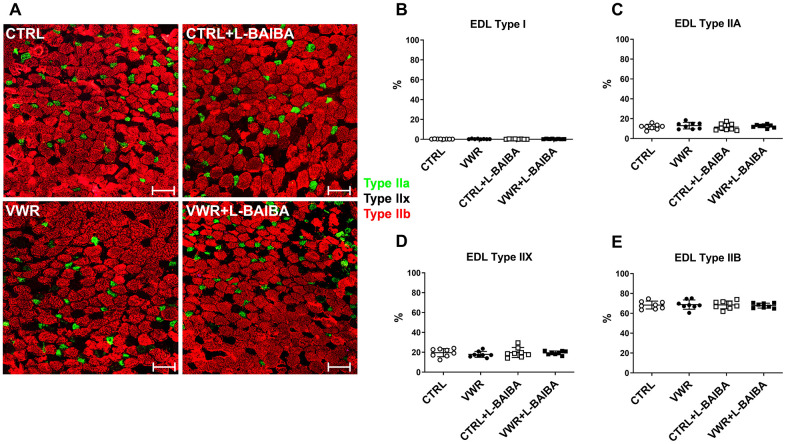
**EDL muscle fiber type analysis after the three-month intervention period.** (**A**) Representative images of EDL muscle histological sections stained for type I (pink), type IIA (green), type IIX (no staining), or type IIB (red) myosin heavy chain. The scale bars represent 100 μm. Quantitation of the percentage of type I (**B**), type IIA (**C**), type IIX (**D**), or type IIB (**E**) myofibers. One-way ANOVA, *p*>0.05. n = 8 CTRL, n = 8 VWR, n = 8 CTRL+L-BAIBA, n = 8 VWR+L-BAIBA. Mean ± SD.

To evaluate the impact of VWR and L-BAIBA supplementation at the level of the individual muscle fibers, histological analyses characterizing the proportions of different muscle fiber types, myofiber number, and the cross-sectional area (CSA) of myofibers were undertaken in stained sections from the soleus and EDL muscles. Based on immunohistochemical staining of skeletal muscle myosin heavy chain isoforms, it was observed that VWR alone modestly increased slow-oxidative type I fibers (48.5 ± 11.2%) and decreased fast-oxidative type IIA fibers (48.2 ± 10.7%) in soleus muscle, which was only statistically significant compared to CTRL+L-BAIBA mice (Type I: 37.5 ± 7.6%, *p*=0.0492; Type IIA: 59.2 ± 7.3%, *p*=0.0347) (Figure 6A–6C). However, the soleus muscles from the mice receiving VWR+L-BAIBA showed a statistically significant increase in the proportion of type I fibers (55.7 ± 7.3%) and decrease in the proportion of type IIA fibers (42.4 ± 7.1%) compared to both CTRL (Type I: 40.2 ± 4.4%, *p*=0.0033; Type IIA: 56.9 ± 4.3%, *p*=0.0040) and CTRL+L-BAIBA groups (Type I: *p*=0.0005; Type IIA: *p*=0.0008) (Figure 6A–6C). There were no significant changes found in the proportions of fast-glycolytic type IIX and IIB fibers in soleus muscle with exercise and/or L-BAIBA supplementation (Figure 6D, 6E). In EDL muscle, no significant differences in the proportions of any of the fiber types were detected among groups (Figure 7A–7E).

**Figure 8:**
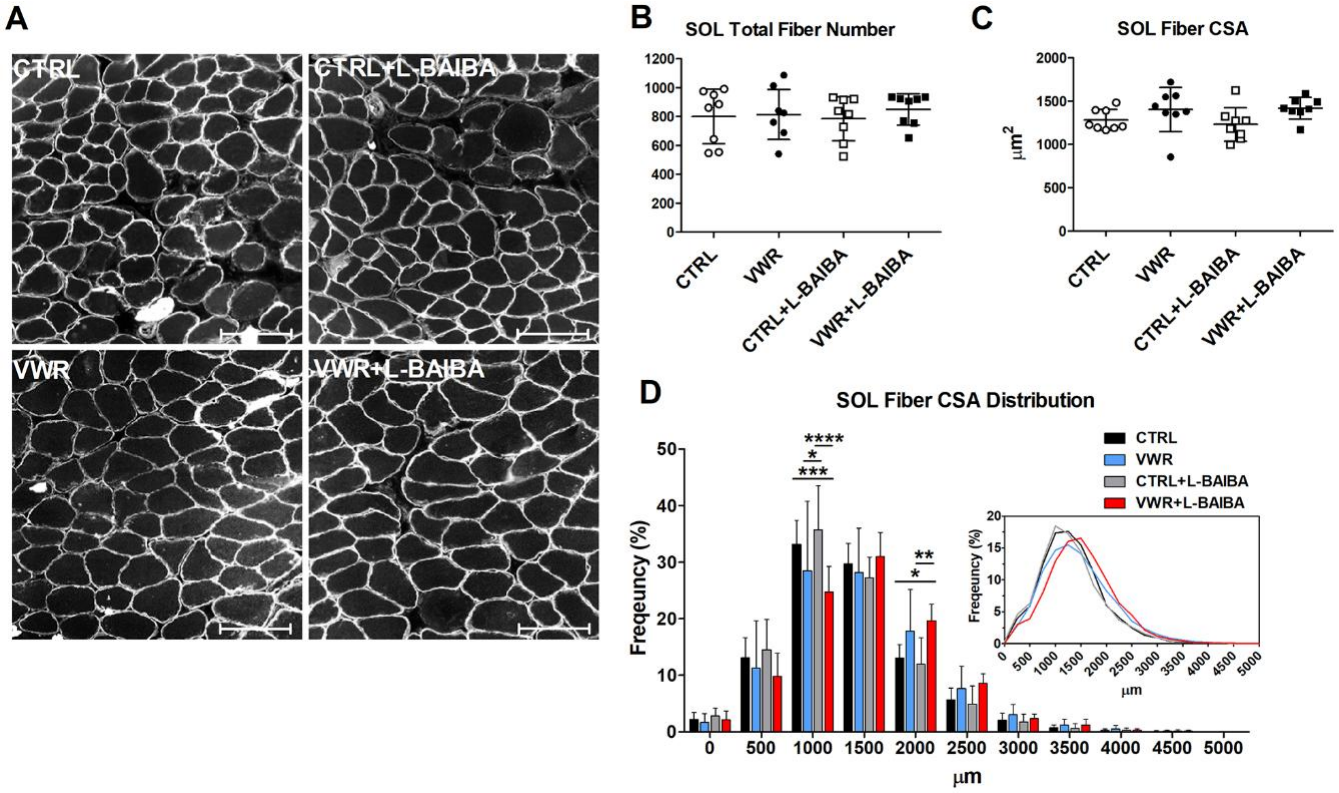
**Soleus muscle fiber cross-sectional area after the three-month intervention period.** (**A**) Representative images of soleus (SOL) muscle histological sections stained for dystrophin for quantitation of myofibers. The scale bar represents 100 μm. (**B**) Quantitation of the total number of SOL myofibers. (**C**) Average SOL myofiber cross-sectional area (CSA). (**D**) The frequency distribution of SOL myofiber CSA. Inlay: Superimposed myofiber frequency distribution curves at 250 μm binning. ****p<0.0001, ***p<0.001, **p<0.01, *p<0.05, Two-way ANOVA with Bonferroni. n = 8 CTRL, n = 8 VWR, n = 8 CTRL+L-BAIBA, n = 8 VWR+L-BAIBA. Mean ± SD.

**Figure 9:**
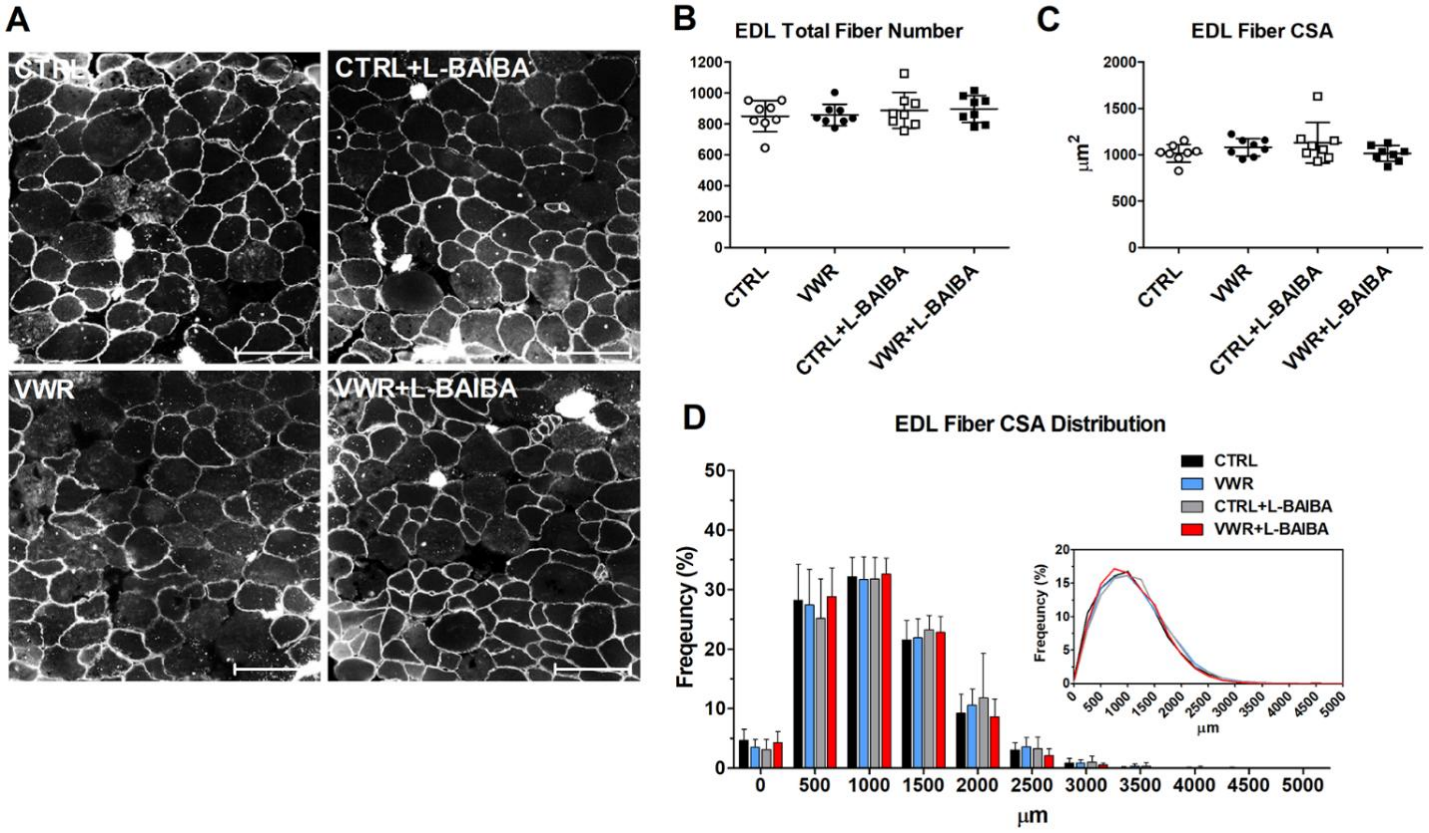
**EDL muscle fiber cross-sectional area after the three-month intervention period.** (**A**) Representative images of EDL muscle histological sections stained for dystrophin for quantitation of myofibers. The scale bar represents 100 μm. (**B**) Quantitation of the total number of EDL myofibers. (**C**) Average EDL myofiber cross-sectional area (CSA). (**D**) The frequency distribution of EDL myofiber CSA. Inlay: Superimposed myofiber frequency distribution curves at 250 μm binning. Two-way ANOVA, *p*>0.05. n = 8 CTRL, n = 8 VWR, n = 8 CTRL+L-BAIBA, n = 8 VWR+L-BAIBA. Mean ± SD.

Next, alterations to the number and size of myofibers were determined (Figure 8A, 8B). The total number of muscle fibers was not significantly altered among any of the interventions assessed in soleus muscle sections (Figure 8B). With regard to muscle fiber size, dystrophin staining did not show statistically significant differences in the average soleus myofiber CSA in mice that underwent VWR or VWR+L-BAIBA compared to CTRL and CTRL+L-BAIBA groups (Figure 8C). However, further analyses of the frequency distribution of myofiber CSA in soleus muscle sections revealed a significant right shift in the size distribution of myofibers in VWR+L-BAIBA muscles. Specifically, VWR+L-BAIBA induced a significant reduction in the proportion of myofibers with a CSA of 1000-1500 μm^2^ (24.7 ± 4.6%) compared to CTRL (33.2 ± 4.2%, *p*=0.0007) and CTRL+L-BAIBA (35.7 ± 7.8%, *p*<0.0001) mice followed by a significant increase in the proportion of myofibers sized 2000-2500 μm^2^ (19.7 ± 2.9%) compared to CTRL (13.1 ± 2.3%, *p*=0.0326) and CTRL+L-BAIBA mice (11.9 ± 4.7%, *p*=0.0032) (Figure 8D). In VWR mice, the soleus CSA distribution had a significant reduction in myofibers with a CSA of 1000-1500 μm^2^ (28.5 ± 12.3%) compared to only CTRL+L-BAIBA mice (*p*=0.0105) with no significant change in the proportion of larger-sized myofibers (Figure 8D). In EDL muscle, the total myofiber number, average fiber CSA, and fiber CSA distributions were not significantly altered in response to VWR and/or L-BAIBA supplementation (Figure 9A–9D).

### Bone properties

Baseline μCT measurements taken prior to the onset of the study showed no significant differences in tibia trabecular parameters among groups (Supplementary Figure 5). To determine the effect of three months of sedentary conditions or VWR with and without L-BAIBA supplementation on bone properties, we conducted μCT analysis and mechanical testing on tibiae (Tables 1, 2). We observed no significant effects on bone structure and mechanical properties in the VWR group. However, we did notice some interesting bone changes when L-BAIBA was supplemented during VWR, including a higher bone volume fraction in the VWR+L-BAIBA group compared to VWR alone, indicating increased cortical bone mass (Table 1). Additionally, VWR+L-BAIBA showed a significantly smaller cortical bone diameter and perimeter than VWR alone (Table 1). For trabecular bone parameters, mice receiving VWR+L-BAIBA displayed higher trabecular thickness compared to CTRL mice (Table 1) and had a smaller trabecular pattern factor than CTRL and VWR mice. Interestingly, the CTRL+L-BAIBA group also showed a smaller trabecular pattern factor compared to CTRL (Table 1).

**Table 1:** μCT analysis of tibiae after the three-month intervention period.

**Cortical bone parameters**	**CTRL**	**VWR**	**CTRL+BAIBA**	**VWR+BAIBA**
Cortical bone mineral density (mg/HA/ccm)	1246 ± 14.32	1241 ± 27.64	1217 ± 31.01	1239 ± 8.60
Cortical bone volume fraction (%)	99.98 ± 0.01	99.94 ± 0.05	99.99 ± 0.02	99.99 ± 0.01^b^
Cortical bone area (mm^2^)	0.69 ± 0.04	0.73 ± 0.06	0.67 ± 0.07	0.67 ± 0.05
Cortical bone thickness (mm)	0.16 ± 0.01	0.17 ± 0.02	0.17 ± 0.01	0.17 ± 0.02
Cortical bone diameter (mm)	2.45 ± 0.16	2.50 ± 0.20	2.31 ± 0.22	2.23 ± 0.12^b^
Cortical bone perimeter (mm)	7.68 ± 0.49	7.84 ± 0.62	7.26 ± 0.68	7.00 ± 0.38^b^
**Trabecular bone parameters**	**CTRL**	**VWR**	**CTRL+BAIBA**	**VWR+BAIBA**
Trabecular bone mineral density (mg/HA/ccm)	988.9 ± 19.65	998.7 ± 11.09	992.1 ± 18.18	1000 ± 21.71
Trabecular bone volume fraction (%)	5.13 ± 1.05	5.32 ± 0.64	4.67 ± 1.80	4.98 ± 0.55
Trabecular bone thickness (mm)	0.035 ± 0.002	0.037 ± 0.002	0.037 ± 0.004	0.039 ± 0.003^a^
Trabecular bone separation (mm)	0.34 ± 0.05	0.33 ± 0.04	0.36 ± 0.06	0.35 ± 0.04
Trabecular bone number (1/mm)	2.97 ± 0.37	3.05 ± 0.40	2.86 ± 0.52	2.86 ± 0.30
Trabecular bone pattern factor (1/mm)	-0.81 ± 0.40	-1.62 ± 1.45	-3.32 ± 1.17^a^	-3.72 ± 1.39^a,b^

^a^*p<0.05* vs. CTRL, ^b^*p<0.0*5 vs. VWR, ^c^*p<0.05* vs. CTRL+L-BAIBA, one-way ANOVA with Tukey. Mean ± SD.

**Table 2:** Mechanical testing of tibiae after the three-month intervention period.

**Cortical bone parameters**	**CTRL**	**VWR**	**CTRL+BAIBA**	**VWR+BAIBA**
Ultimate load (N)	15.72 ± 2.68	15.56 ± 2.48	14.55 ± 1.49	16.10 ± 1.64
Elastic stiffness (N/mm)	65.92 ± 7.97	65.66 ± 8.03	59.12 ± 3.63	62.26 ± 7.55
Total work to failure (mJ)	3.70 ± 1.12	3.45 ± 2.19	2.52 ± 0.32	4.01 ± 1.28
Elastic work to failure (mJ)	1.74 ± 0.31	1.76 ± 0.49	2.06 ± 0.45	1.79 ± 0.67
Plastic work to failure (mJ)	1.97 ± 1.11	1.70 ± 2.05	0.46 ± 0.21	2.22 ± 1.82
Elastic displacement (mm)	0.24 ± 0.03	0.23 ± 0.04	0.26 ± 0.03	0.23 ± 0.04
Post-yield displacement (mm)	0.14 ± 0.07	0.11 ± 0.13	0.03 ± 0.02	0.18 ± 0.17
Modulus of elasticity (Gpa)	3.82 ± 0.24	4.13 ± 0.43	4.78 ± 0.36^a,b^	4.77 ± 0.36^a,b^
Moment of inertia (mm^4^)	0.26 ± 0.03	0.24 ± 0.02	0.19 ± 0.02^a,b^	0.20 ± 0.02^a,b^

^a^*p<0.05* vs. CTRL, ^b^*p<0.0*5 vs. VWR, one-way ANOVA with Tukey. Mean ± SD.

Next, biomechanical properties of the tibiae were assessed using a three-point bending procedure. The results of the mechanical testing revealed that both VWR+L-BAIBA and CTRL+L-BAIBA groups demonstrated a significantly larger modulus of elasticity but a smaller moment of inertia than CTRL and VWR (Table 2). No differences were found between the mechanical parameters of ultimate load, elastic stiffness, work to failure, or displacement among groups (Table 2).

Lastly, the adipose content of the femur was assessed by histomorphometry showing the bone marrow adipose tissue area significantly decreased with VWR+L-BAIBA treatment compared to CTRL mice, while there was no change in bone marrow adipose tissue area with VWR alone (Table 3).

**Table 3:** Quantification of bone marrow adipose tissue after the three-month intervention period.

**Bone marrow parameter**	**CTRL**	**VWR**	**CTRL+BAIBA**	**VWR+BAIBA**
Bone marrow adipose tissue area (mm^2^)	0.08 ± 0.03	0.08 ± 0.06	0.06 ± 0.05	0.03 ± 0.03^a^

^a^*p<0.05* vs. CTRL, one-way ANOVA with Tukey. Mean ± SD.

## DISCUSSION

In this study we hypothesized that VWR combined with L-BAIBA supplementation for three months would promote greater cardiac and musculoskeletal benefits than VWR alone. In summary, we found that, while L-BAIBA supplementation had no effect alone in the heart or skeletal muscle, the combination of VWR+L-BAIBA altered cardiac ECG and resulted in significant muscle hypertrophy and augmentation of contractile function in the slow-twitch soleus muscle. At the myofiber level, VWR+L-BAIBA increased type I slow-oxidative myofiber content and shifted the myofiber size distribution towards larger fibers in the slow-twitch soleus muscle, while VWR alone did not have a significant impact on these characteristics compared to CTRL mice. On the other hand, EDL muscle showed improved fatigue resistance and increased caffeine-mediated force recovery from fatigue with VWR alone, but not with the combination of VWR+L-BAIBA, suggesting L-BAIBA preferentially improves slow-twitch muscle adaptation to exercise while maintaining the original properties of fast-twitch muscle. Lastly, L-BAIBA alone and VWR+L-BAIBA, but not VWR alone, improved material and structural properties of bone.

The interaction of muscle, bone, and the heart during exercise is multifaceted, involving mechanical coupling as well as mutual biochemical signaling through secreted molecules, the latter emerging as a promising novel target for therapeutic intervention. Adaptation of musculoskeletal and cardiac tissue to exercise remains incompletely understood, especially during the aging process. The muscle-derived exercise metabolite, L-BAIBA, has been found to be important for transducing many beneficial effects associated with regular exercise, such as regulation of metabolism, alleviation of inflammation, and protection against oxidative stress in several organs including musculoskeletal and cardiac tissue. Here, we describe a novel function for L-BAIBA, showing that L-BAIBA supplementation synergizes in combination with regular endurance exercise to enhance slow-twitch soleus skeletal muscle hypertrophy, contractile strength, fiber type switching, and bone structural and material qualities. Regular exercise is the best-known intervention to maintain the quantity and quality of musculoskeletal tissue and to counteract its aging-associated decline that begins to manifest after middle age [9]. As we have previously shown that L-BAIBA supplementation mitigates the deterioration of musculoskeletal tissue during the catabolic state of prolonged disuse [25], our findings that L-BAIBA can also promote musculoskeletal benefits under the anabolic stimulus of exercise further expands its therapeutic potential.

### Heart properties

Regular endurance exercise has been found to be associated with structural remodeling and altered functional properties of the heart [28–30]. Through conscious ECG monitoring, it was found that many of the ECG parameters including heart rate, HRV, PR interval (atrioventricular coupling) and QRS interval (ventricular depolarization) were not significantly altered with L-BAIBA treatment with and without VWR. However, the combination of VWR with L-BAIBA supplementation, but not VWR alone, induced a modest prolongation of the QTc interval by 4% compared to CTRL mice, indicating a longer ventricular repolarization time. Some degree of QTc prolongation is considered normal with exercise as shown in previous studies of human athletes and animal models of exercise and is typically accompanied by increased ventricular mass [31–33]. Interestingly, there were no significant differences observed in heart weight or HW/BW among groups in the present study. Nevertheless, these results are in line with those in soleus muscle and bone displaying a potentiating effect of L-BAIBA supplementation on the exercise response in the heart. Further studies are still needed to fully understand the effects of VWR+L-BAIBA on heart physiology.

### Muscle properties

Aging skeletal muscle is characterized by reduction of myofiber CSA and contractile force production [34], which can negatively affect daily physical activity and mobility. We found that supplementation with L-BAIBA during voluntary endurance exercise for three months in middle-aged mice promoted anabolic changes greater than exercise alone, including hypertrophy of the slow-twitch soleus muscle as evidenced by greater average muscle weight and a rightward shift in the distribution of myofiber CSA towards larger size fibers in VWR+L-BAIBA mice but not with VWR alone. These findings indicate an enhanced hypertrophic response for soleus muscle when running exercise is combined with L-BAIBA supplementation. Importantly, L-BAIBA combined with exercise improved muscle functional capacity showing gains in contractile strength, especially at submaximal contractile force, which represents the physiologically relevant functional range for mammalian skeletal muscle [35, 36]. The reduced overall effectiveness of VWR alone on muscle size and force in this study may be due to the age of the mice, since these muscle properties typically peak around middle age before the onset of age-related mass and strength decline [37]. Nevertheless, the results of our VWR intervention are consistent with another study showing no significant changes to soleus or gastrocnemius muscle size or myofiber CSA following 10 weeks of low-resistance VWR in middle-aged mice [38]. The improvements in muscle, when exercise was combined with L-BAIBA, were present even though there was no added wheel resistance, and animals receiving L-BAIBA ran the same daily distance as those that were not supplemented over the intervention period, suggesting that L-BAIBA improved the effectiveness of exercise training on promoting muscle mass and strength gains. Thus, L-BAIBA supplementation may be useful for maintaining muscle tissue abundance during aging or for enhancing the anabolic effects of endurance exercise in those with sarcopenia.

L-BAIBA supplementation combined with exercise was found to increase the proportion of soleus muscle slow-oxidative myofibers that express MHC type I by +15.5% compared to controls, while VWR alone had less of an impact on the number of type I myofibers (+8.2%) which was not statistically significant compared to control. This adaptation towards slow type I myofibers in soleus in response to exercise combined with L-BAIBA was also accompanied by a significant -14.5% reduction in fast-oxidative type IIA myofibers. The enhanced ability of VWR+L-BAIBA to influence muscle fiber type proportions over VWR alone suggests that L-BAIBA may potentiate the effects of endurance exercise on promoting a transition to a slow-twitch fiber type and oxidative metabolic profile in the soleus. The fiber type changes towards higher type I content in soleus muscle are consistent with the changes in contractility observed from the *ex vivo* muscle functional studies. A leftward shift of the soleus force-frequency relationship was seen in mice that received VWR+L-BAIBA compared to both VWR alone and control, a characteristic of increased type I myofiber content in skeletal muscle. These findings indicate that a lower threshold of electrical stimulation was required in VWR+L-BAIBA muscles to produce similar levels of submaximal force as CTRL, CTRL+L-BAIBA, and VWR mice. Type I fibers harbor enhanced calcium sensitivity relative to fast-twitch isoforms, allowing for greater amounts of contractile force at lower frequencies of stimulation [37]. As such, L-BAIBA combined with exercise favored enhancements to soleus muscle submaximal force production (+27%), as the improvements were nearly 2-fold greater than for maximal force (+15%).

The potentiating effects of L-BAIBA treatment on primarily slow-oxidative muscle size and function in response to exercise could be due to enhanced activation of exercise signaling pathways. L-BAIBA is derived from metabolism of branched chain amino acid, valine, and supplementation of branched chain amino acids (leucine, isoleucine, valine) and their metabolites have previously been found to harbor potential in improving muscle hypertrophy and strength, especially when combined with a regular exercise intervention through activation of the mTOR signaling pathway leading to increases in protein synthesis [39, 40]. Like other branched chain amino acid metabolites, L-BAIBA signaling may also target the mTOR pathway as previous studies in several different tissue types including skeletal muscle have shown that BAIBA enhances expression and phosphorylation of AKT [41–43], an important positive regulator of mTOR activity. Conversely, signaling pathways involved in regulation of muscle protein degradation and atrophy may have been downregulated by L-BAIBA supplementation with exercise leading to augmented soleus muscle mass and strength include those involving FOXO transcription factors which mediate the muscle catabolic state through the control of E3 ubiquitin ligases atrogin-1 and muscle ring finger-1 [44, 45]. Next, endurance exercise is typically associated with metabolic and muscle fiber type switching towards a more slow-twitch oxidative profile to accommodate the elevated activity and metabolic demand. Increased mitochondrial respiration/abundance and enhanced metabolic control are common cellular adaptations occurring during and following endurance exercise. These adaptations are largely regulated by the energy sensor AMPK that is activated by accumulating AMP generated as ATP levels become depleted during repetitive muscle contraction [46]. Numerous studies have found that many of the positive effects of BAIBA are mediated through the AMPK pathway leading to improvements in metabolic substrate utilization and protection against metabolic stress [23, 26, 41]. An important downstream target of AMPK activation is the phosphorylation of PGC-1α [47], a master transcriptional regulator of metabolic capacity, leading to enhanced mitochondrial biogenesis and stimulation of oxidative gene expression including cytochrome oxidase II, ATPase and cytochrome C [48]. PGC-1α is also important in the regulation of slow fiber gene expression and fiber type determination in skeletal muscle by exercise through co-activation of Mef2 transcription factors and the calcineurin signaling pathway [48]. BAIBA has also been found to stimulate PPARδ signaling in skeletal muscle [41], another mediator of the slow-twitch phenotype [49]. Thus, L-BAIBA supplementation may synergize with endurance exercise to promote enhanced activation of anabolic pathways and activate slow-oxidative muscle fiber type transitioning in skeletal muscle.

Enhancement of the slow-oxidative muscle fiber phenotype when L-BAIBA is combined with exercise may have important therapeutic applications for a variety of disorders. This includes aiding the recovery of muscular function following prolonged unloading conditions (e.g., bed rest, spinal cord injury, spaceflight) that is traditionally characterized by atrophy of specifically slow-twitch myofibers and a fiber type transition from type I oxidative to type II glycolytic myofibers [50, 51]. In addition, metabolic diseases such as obesity and type 2 diabetes mellitus are associated with a downregulation of slow type I muscle fibers, and an increase in fast type II fiber content in skeletal muscle [52, 53]. Thus, increased type I myofibers are associated with improved metabolic health and reduced adiposity. In line with this, bone marrow adipose content of the VWR+L-BAIBA animals was significantly reduced by 63% compared to sedentary controls, while the average amount of adipose in the bones of VWR animals was similar to sedentary control levels.

The EDL muscle was affected differently by endurance exercise and L-BAIBA supplementation. In agreement with previous wheel running studies from our group and others, EDL muscle mass and contractile strength were not altered by voluntary endurance exercise, as opposed to the soleus muscle [54–56]. Instead, VWR alone resulted in a small enhancement to fatigue resistance during the early phase of muscle fatigue that was not evident in mice with VWR+L-BAIBA treatment. Next, although there were no differences between groups in the physiological recovery period following fatigue, VWR mice showed increased potentiation of contractile force in response to caffeine treatment at the end of the recovery period, but the VWR+L-BAIBA group did not display this change. As L-BAIBA treatment by itself did not produce any noticeable changes in EDL muscle size or function this suggests that L-BAIBA may have altered the effects of endurance exercise on fast-twitch muscular fatigue and recovery with caffeine. However, there were no changes observed at the EDL myofiber level among groups that could corroborate these functional differences. Additionally, physical running capacity was unaffected between groups that exercised with and without L-BAIBA. It is possible that L-BAIBA may have altered changes in EDL muscle calcium handling with exercise since caffeine treatment induces release of intracellular calcium stores [57] and was administered at the end of the contractility experiments to gauge maximal calcium-related recovery of muscle force after fatigue. Alternatively, metabolic switching from glucose to predominantly fatty acid-based fuel sources due to BAIBA treatment may have also contributed to the results in EDL. Previous studies have shown that metabolic switching, for example due to chronic PPARδ stimulation, can decrease muscle functional performance during repetitive isometric contractions that require rapid utilization of glucose for ATP production [58].

### Bone properties

We found that VWR alone for three months was not sufficient to impact bone tissue properties in middle-aged male mice. Remarkably, supplementation with L-BAIBA significantly improved both cortical and trabecular bone parameters compared to sedentary and exercising mice. VWR+L-BAIBA supplementation maintained higher cortical bone volume fraction, trabecular bone thickness, and connectivity. The cortical bone volume fraction measured in this study does not include the bone marrow area, so it is considered to reflect the amount of cortical porosity. Although these changes were not substantial, L-BAIBA supplementation may help prevent the accumulation of cortical porosity and maintain trabecular bone connectivity by preserving thickness during endurance exercise in aging animals. Trabecular bone connectivity affects cortical bone strength by preventing the accumulation of microdamage by diffusing the load into the trabecular network [59]. A well-developed trabecular network is associated with higher bone strength [60]. Additionally, cortical porosity negatively correlates with the modulus of elasticity [61]. Therefore, these alterations could contribute to maintaining bone strength.

To evaluate the effects of VWR and L-BAIBA supplementation on bone strength, we conducted mechanical testing on the tibiae. Our results revealed that the VWR+L-BAIBA group exhibited a lower moment of inertia, a geometric property that predicts bone strength in bending, compared to both the CTRL and VWR groups, potentially due to a smaller bone diameter. However, we also observed that the VWR+L-BAIBA group displayed a higher modulus of elasticity, a material property that measures the stiffness of a material. This suggests that, despite the prediction of weaker bone strength based on the moment of inertia, the bone in the VWR+L-BAIBA group is structurally stiffer and more resistant to elastic deformation. Interestingly, this effect on bone mechanical properties was primarily attributed to the L-BAIBA supplementation itself, rather than the accompanying exercise. L-BAIBA treatment alone produced similar changes in bone properties as the VWR+L-BAIBA combination, suggesting that L-BAIBA supplementation, with and without endurance exercise, may help preserve bone material properties during aging. As a result, the tibiae in these groups may require less periosteal expansion to compensate for the impaired material properties typically associated with aging, which led to the smaller bone diameter in these groups.

Several recent studies, including the results from this study, suggest that skeletal anabolic pathways are a significant target of muscle-secreted factors. Conditioned media collected from *ex vivo* contracting skeletal muscles enhanced the activation of the PI3K/Akt and β-catenin pathways compared to media from non-contracted muscles when applied to murine osteocytes undergoing fluid flow sheer stress, implicating a role for muscle factors, like L-BAIBA, in improving the response to loading [62]. Furthermore, a recent report by Prideaux et al. found that L-BAIBA supplementation in mice over two weeks increased the effectiveness of a sub-optimal mechanical loading protocol [27]. Importantly, the sub-optimal loads applied in this study were, alone, not sufficient to significantly alter bone properties, but when mice were treated with L-BAIBA, sub-optimal loading promoted endosteal and periosteal bone formation and induced gene transcriptional changes in bone, specifically to the insulin-like growth factor receptor signaling pathway, including insulin-like growth factor binding protein-2 (*Igfbp2*) which is involved in the regulation of bone mass [63, 64]. In a similar vein, the results from the present study show that VWR alone for three months was not a sufficient stimulus for increasing bone content and structural properties in male mice, while the addition of L-BAIBA promoted a positive adaptational response to physical activity, suggesting that L-BAIBA may lower the threshold of active loading required during exercise to promote maintenance of bone during aging.

Bone marrow adipose tissue was significantly decreased in the VWR+L-BAIBA group, but not with VWR alone. BAIBA promotes the browning of white adipose tissue through PPARα [22]. These beige adipocytes express uncoupling protein 1 (UCP1), which disrupts oxidative phosphorylation in mitochondria, diverting energy from ATP production to heat generation [65]. This thermogenic process significantly increases energy expenditure, contributing to fat loss. Furthermore, BAIBA reduces fat accumulation by upregulating genes involved in fatty acid oxidation while downregulating lipogenic genes [22, 23]. The reduction of bone marrow adipose tissue observed in the VWR+L-BAIBA group may be attributed to similar mechanisms of action exerted by L-BAIBA. Free fatty acids liberated from bone marrow adipose tissue induced by L-BAIBA may serve as a potential energy source for bone cells such as osteoblasts [66, 67], thereby supporting their metabolic activity and function during exercise.

### Limitations

There are some limitations contained within this study that should be considered in the interpretation of the results. We showed that baseline measurements of bone trabecular parameters and animal body weights prior to the beginning of the study were similar among groups but baseline characterization of bone cortical and skeletal muscle parameters was not performed. Therefore, it is possible that undetected baseline differences may have contributed to the study results in cortical bone and skeletal muscle. Next, our grouping strategy to allocate mice to CTRL/CTRL+L-BAIBA or VWR/VWR+L-BAIBA groups was based on propensity to utilize the exercise wheel, so we cannot fully exclude differences in underlying animal health between CTRL/+L-BAIBA and VWR/VWR+L-BAIBA groups that may have influenced the study outcomes. Another limitation of the study was that the animal population consisted of only male mice. There are inherent differences in cardiac and musculoskeletal adaptation to endurance exercise training among males and females [29, 68, 69], which may therefore influence the physiological outcomes of L-BAIBA supplementation during exercise. Next, the exercise regimen administered in this study consisted of voluntary running without the use of added resistance. In the future it will be important to determine how L-BAIBA supplementation affects the musculoskeletal adaptation to different forms of exercise, such as resistance training or the combination of endurance with resistance training. We used 12-month-old male mice as a model of middle age. It would be of interest to determine if the changes we observed would persist into older age and to explore the effects of beginning these interventions in older age mice. Lastly, we specifically utilized the L-isomer of BAIBA in this study based on our previous work, but recent studies have shown that D-BAIBA levels in the circulation can also be stimulated by exercise and can regulate bone physiology [70, 71]. Thus, further studies on the effects of D-BAIBA during exercise are warranted.

In conclusion, the findings of this study indicate a physiological interaction between endurance exercise and L-BAIBA supplementation to enhance soleus muscle mass, strength, type I myofiber content, bone structural and material qualities, and prevent an increase in bone marrow adipose content. The combination of L-BAIBA with exercise may be efficacious for improving anabolic aspects of exercise training and metabolic health in musculoskeletal tissues during aging.

## MATERIALS AND METHODS

### Animals

All animal studies were approved by the UMKC Institutional Animal Care and Use Committee (IACUC) in accordance with animal welfare guidelines (protocol # 1301). Ten to eleven-month-old C57Bl6 male mice were obtained from the NIH/NIA aging colony via Charles Rivers Laboratories. All of the mice were given access to a voluntary wheel running (VWR) apparatus (Mouse Home Cage Running Wheel, Columbus Instruments, Columbus, OH, USA) in their cages for 1-2 wk, to determine which mice had a tendency to run. Each wheel has a magnetic indicator and a Hall effect sensor that connects to a computer interface and records wheel revolutions using the Multi-Device Interface MDI software (Columbus Instruments). The data was exported into a CSV format at the end of the experiment.

At 12 months of age, mice were provided access to wheels for a 1-week conditioning period to determine interest in using the running wheel. The mice that showed the highest running activity were then divided randomly into either the voluntary wheel running exercise group (VWR) or the VWR in combination with L-BAIBA supplementation groups (VWR+L-BAIBA), Age and body weight-matched mice that were not allocated to either of the VWR groups were randomly divided into the control group (CTRL) or control with L-BAIBA supplementation group (CTRL+L-BAIBA). The CTRL and CTRL+L-BAIBA groups were placed in standard mouse cages and housed under normal conditions, while the VWR and VWR+L-BAIBA groups were placed in standard mouse cages containing the voluntary wheel running apparatus. VWR and VWR+L-BAIBA mice had unrestricted access to the wheel for 3 mo (13 weeks), except during week 6 in which wheel running was suspended for one week. This rest period was inserted to encourage renewed wheel running activity in male mice. All mice were maintained on a 12 hr light/dark cycle at 22° C constant temperature and 45-55% humidity with standard lab chow ad libitum, before sacrificing at 15 mo of age. For supplementation with L-BAIBA, L-BAIBA (100 mg/kg/day, AdipoGen Life Sciences) was provided in drinking water ad libitum. All mice were individually housed throughout the experiment, monitored daily and weighed monthly, and were provided food and water ad libitum, in accordance with an approved IACUC protocol (UMKC IACUC Protocol 1301). On the day of sacrifice, final weights and a health report were recorded. Two independent experiments were performed at separate times for the CTRL/VWR mice and the CTRL+L-BAIBA/VWR+L-BAIBA mice, during April 2021 and November 2021, respectively. Final sample sizes were n=8-9 mice per group.

### Electrocardiogram and cardiac tissue isolation

*In vivo* conscious electrocardiogram (ECG) was measured in 15 mo old CTRL, CTRL+L-BAIBA, VWR, and VWR+L-BAIBA mice after completion of 3 months of VWR or sedentary conditions using a non-invasive ECGenie apparatus (Mouse Specifics, Boston, MA, USA). Mice were first placed on a platform outside the apparatus and allowed a five-minute acclimation period. Next, mice were allowed to enter the ECG measurement compartment where ECG signals were recorded via the paws through electrodes in the floor. ECG signals were collected over a 20-minute time period and analyzed using Emouse software (MouseSpecifics).

Whole hearts were isolated from mice after sacrifice by cervical dislocation and placed in a dish containing Hank’s balanced salt solution. Next, the heart was cut transversely and blood and excess tissues were removed. The heart was then weighed for determination of heart weight and heart weight to body weight ratio (HW/BW) and flash frozen in liquid nitrogen and stored at -80° C.

### *Ex vivo* skeletal muscle contractility

15 mo old CTRL, CTRL+L-BAIBA, VWR, and VWR+L-BAIBA mice were sacrificed by cervical dislocation, and the soleus and extensor digitorum longus (EDL) muscles were removed for contractility analysis as previously described [54]. Following mounting of muscles to the contractility system (GlobalTown Microtech, Sarasota, FL, USA) contractile analysis was performed in physiological buffer (144 mM NaCl; 5 mM KCl; 1 mM MgCl_2_; 25 mM NaHCO_3_; 2.5 mM CaCl_2_; 10 mM glucose; pH 7.45) maintained at 37° C and aerated with 95%/5% O2/CO2. Soleus and EDL muscles were first allowed a 30 min equilibration period during which time they were contracted with maximal and submaximal-frequency stimulations with a three-minute rest interval (160/40 Hz for soleus; 200/100 Hz for EDL). Following equilibration, soleus and EDL muscles were stimulated to contract with frequencies ranging from 1-220 Hz to generate the force-frequency relationship. Next, physiological buffer was replaced with a calcium-depleted physiological buffer (144 mM NaCl; 5 mM KCl; 1 mM MgCl_2_; 25 mM NaHCO_3_; 0 mM CaCl_2_; 0.1 mM EGTA; 10 mM glucose; pH 7.45) and soleus and EDL muscles were contracted with alternating maximal and submaximal-frequency stimulations with a three-minute rest interval for 30 min. The buffer was then replaced with physiological buffer with normal calcium concentration, and muscles were allowed to recover under continued maximal and submaximal contractions for 30 min. To induce fatigue, the muscles were next contracted with alternating maximal and submaximal stimulations for 5 min with a rest interval of two seconds. Immediately following the fatiguing protocol, muscles were allowed a 30 min recovery period while contracting at maximal and submaximal force with a rest period of 3 min, followed by 5 mM caffeine addition to the muscle contractility chambers to evaluate calcium availability during the recovery period. At the end of the experiment, soleus and EDL muscle optimal length and muscle weight were measured, and left limb soleus and EDL muscles were snap frozen in liquid nitrogen and stored at -80° C while right limb soleus and EDL muscles were frozen in Tissue-Tek O.C.T. compound (Sakura Finetek USA Inc., Torrence, CA, USA) in liquid nitrogen-cooled isopentane and saved at -80° C for histological analysis. A PowerLab/LabChart Software system (ADInstruments, Colorado Springs, CO, USA) was used to store and analyze force data. Muscle force is reported as absolute force (mN) and force normalized to the muscle physiological cross-sectional area (N/cm^2^) via the following formula:

$$\frac{\begin{array}{c} (force\;(mN)*muscle\;\\ length\;(mm)*1.06\;\\ \left(mg\text{/}mm^{3}\;muscle\;density)\right) \end{array}}{muscle\;weight\;(mg)}*\;0.1\;(conversion\;factor)$$

### Assessment of skeletal muscle fiber type and cross-sectional area (CSA)

Muscles were retrieved from the contractility experiments, excess buffer solution removed, and muscle placed in Tissue-Tek O.C.T. compound before transfer to a Cryomold. The muscle was oriented into its natural shape by gently pulling the ends of tendon, and the Cryomold was snap frozen in isopentane in liquid nitrogen. 7 μm thick transverse cryosections were cut using a Leica CM3050S cryomicrotome (Leica Microsystems, Wetzlar, Germany) from four standardized levels through the muscle, at 750 μm intervals between levels, with three sections cut per level.

Muscle fiber typing was determined as previously described using a 4-color immunofluorescent staining method using antibodies specific for myosin heavy chains (MyHC) corresponding to the different fiber types [54]. These antibodies included: Mouse IgG2b BA-D5 specific for MyHC I (1:100, Developmental Studies Hybridoma Bank (DSNB), Iowa City, IA, USA), mouse IgG1 SC-71 specific for MyHC IIA (1:100, DSHB), mouse IgM BF-F3 specific for MyHC IIB (1:20, DSHB). In addition, we used a rabbit anti-dystrophin antibody (1:400, ab15277, Abcam, Cambridge, England) to label the border of each fiber. The muscle cryosections were air dried for 5 min and fixed with acetone for 5 min at room temperature. They were then blocked to eliminate non-specific binding in M.O.M ® Blocking Reagent (1:25, MKB-2213, Vector Laboratories, Inc., Burlingame, CA, USA) for 1 hr at room temperature. The sections were then incubated with primary antibodies specific for each MyHC as described above, along with anti-dystrophin antibody overnight at 4° C. After washing in phosphate-buffered saline (PBS), the sections were incubated with the secondary antibodies for 2 hr at room temperature. These included: an Alexa Fluor 647 goat anti-mouse IgG2b secondary antibody (A21242, Invitrogen, Carlsbad, CA, USA) for BA-D5, an Alexa Fluor 488 goat anti-mouse IgG1 secondary antibody (A21121, Invitrogen) for SC-71, an Alexa Fluor 594 goat anti-mouse IgM secondary antibody (A21044, Invitrogen) for BF-F3, an Alexa Fluor 350 goat anti-rabbit IgG (H+L) secondary antibody (A11046, Invitrogen) for dystrophin. After further washing in PBS and post-fixation with methanol, sections were coverslip mounted using vector shield mounting media (H-1000, Vector Laboratories, Inc.). For analysis of fiber size, sections were immunostained singly with the rabbit anti-dystrophin antibody as above but using a CF555 goat anti-rabbit IgG (H+L) secondary antibody (20232, Biotium, Fremont, CA, USA).

The fluorescent images for fiber type and cross-sectional area (CSA) measurements were acquired using a Keyence BZ-X810 microscope using a 10x. 45NA objective and excitation and emission filters appropriate for each fluorescence dye. Pseudocoloring and merging of multiplexed images were performed by a BZ-X800 analyzer. To quantify the number of each fiber type, the Multi Point counting tool was used in the Image J software (Rasband, W.S., ImageJ, U. S. National Institutes of Health, Bethesda, MD, https://imagej.nih.gov/ij/, 1997-2018). The skeletal muscle fibers that were not stained with any antibodies were counted as Type IIX.

For muscle fiber cross-sectional area (CSA) analysis, images of fiber cross-sections were first processed in Fiji. Contrast normalization and a 2-pixel Gaussian blur were applied to enhance the continuity of dystrophin-defined borders while preserving fiber boundary details. No specialized plugins were used.

Next, the dystrophin-stained mouse muscle cross-sections were analyzed in Ilastik (https://ilastik.org) [72] using its Autocontext pixel classification workflow. Four pixel classes were defined: (1) background, (2) dystrophin-labeled, (3) fiber interior, and (4) immunofluorescence labeling artifact. A parallel random forest classifier (Vision with Generic Algorithms, VIGRA) was trained with limited annotations on a subset of ten randomly chosen images. Once trained, this pixel classifier generated pixel probability maps for all images based on each pixel’s likelihood of belonging to one of the four classes.

These probability maps were then imported back into Ilastik for object segmentation, object classification, and fiber area measurement. A second parallel random forest classifier (VIGRA) was employed to classify segmented objects using the full complement of object shape and object intensity measurements available in Ilastik. Training focused on excluding improperly segmented objects caused by incomplete dystrophin labeling (where multiple fibers might appear as one object), highly irregular fiber object shapes, objects where dystrophin labeling around fibers was saturated and created an artificial shrinkage of fiber cross-sectional area, and extremely small and poorly defined object segmentations. The resulting object classifier was applied to all segmented images, producing a collection of accurately segmented, dystrophin-defined, fiber cross-sectional areas. Cross-sectional area measurements were then collected from each fiber object and tabulated for further analysis. Two serial sections were examined per muscle sample for CSA and the results were averaged.

### Adipose analysis by histomorphometry

The distal femur was fixed in 4% paraformaldehyde in PBS, pH 7.4 for 24 hr, then transferred to 70% ethanol. Undecalcified distal femurs were then dehydrated in a graded series of alcohols. They were then infiltrated with acetone, then 1:1 followed by 1:2 acetone:methyl methacrylate infiltration solution [84% methyl methacrylate (MMA), 14% dibutyl phthalate, 1% polyethylene glycol, 0.7% benzoyl peroxide], and then two changes of 100% MMA infiltration solution (solutions changed daily). Samples were then embedded in a 20 ml glass vial containing 5 ml pre-polymerized base by adding 10 ml freshly made MMA embedding solution [as above for the infiltration solution but with benzoyl peroxide reduced to 0.4% and with 0.33% N,N-dimethyl p-toluidine]. Polymerization was done at -20° C for 3-5 days.

Blocks were trimmed with a Buehler ISOMET 1000 precision saw, then 5 μm longitudinal sections were cut on a Thermo Scientific Microm HM355S microtome, using a Dorn and Hart microedge Tungsten carbide D profile knife. After the sections were deplasticized and rehydrated, they were stained with Goldner’s stain using standard histological procedures and marrow adipose tissue was quantified using a 10x objective with a Nikon E800 microscope (Nikon Instruments, Inc. Melville NY, USA) and SONY Exwave HAD camera (Sony Corp., New York, NY, USA) interfaced with an Osteomeasure bone histomorphometry system (OsteoMetrics, Decatur, GA, USA). Bone marrow adipose percent area was measured in the entire metaphyseal region below the growth plate and extending distally 550 μm below the lowest point of the growth plate. Values were averaged from 3 non-adjacent sections per animal for each data point.

### Micro-CT analysis

Mouse tibiae were scanned in the Bruker Skyscan 1174 (Billerica, MA, USA) at a nominal resolution of 9.6 microns employing an aluminum filter 0.5 mm thick and an applied x-ray tube voltage of 50 kV. Camera pixel binning was not applied. The scan orbit was 180/360 degrees with a rotation step of 0.4 degrees. Reconstruction was carried out with a modified Feldkamp algorithm using the SkyScanTM NRecon software accelerated by GPU3. Gaussian smoothing, ring artifact reduction, and beam hardening correction were applied.

The stack of the reconstructed images was analyzed using CTAn software. Fifty slices (0.5 mm) from the mid-diaphysis were selected for each tibia to calculate the cortical bone parameters. The minimum threshold was set to 80 and the maximum to 255 for all the bones. One hundred slices (1 mm) from the growth plate of distal tibia were selected to calculate the trabecular bone parameters.

### Three-point bending test

Three-point bending was performed on tibias that had been freshly frozen in PBS-soaked gauze at sacrifice. The samples were thawed at least half an hour before testing was started. All tests were performed on a Bose 3230 Dynamic Loading system using a 225 N load cell and Wintest 4.0 software. Prior to testing, the length, minimum and maximum diameter and weight were measured for each sample. Tibias were tested while keeping the posterior - anterior axis in the loading direction and the lateral - medial axis in the bending direction. All samples were centered on the supports, and the force was applied vertically to the midshaft at a constant speed of 0.1 mm/sec until fracture occurred. Displacement and load data were collected for biomechanical analysis to obtain bone properties using an ImageJ plugin, BoneJ [73].

### Statistical analyses

For comparisons between the four groups of normally distributed data one-way analysis of variance (ANOVA) was used followed by Tukey’s post hoc test. Kruskal Wallis was used to analyze datasets that were not normally distributed with Dunn’s post hoc test. Two-way ANOVA with Bonferroni was used to compare the mean differences between groups split by two independent variables. A *p*-value <0.05 was considered statistically significant. All data are presented as mean ± standard deviation (SD).

## Supplementary Material

Supplementary Figures
