# Supplementary material for: L-β-aminoisobutyric acid (L-BAIBA) in combination with voluntary wheel running exercise enhances musculoskeletal properties in middle-age male mice
Source: Aging (Albany NY). 2025 Oct 1;17(10):2475–99. doi: 10.18632/aging.206325 (PMC12606963; doi:10.18632/aging.206325)
Supplement: Supplementary Figures [file aging-17-10-206325-s001.pdf]

SUPPLEMENTARY FIGURES

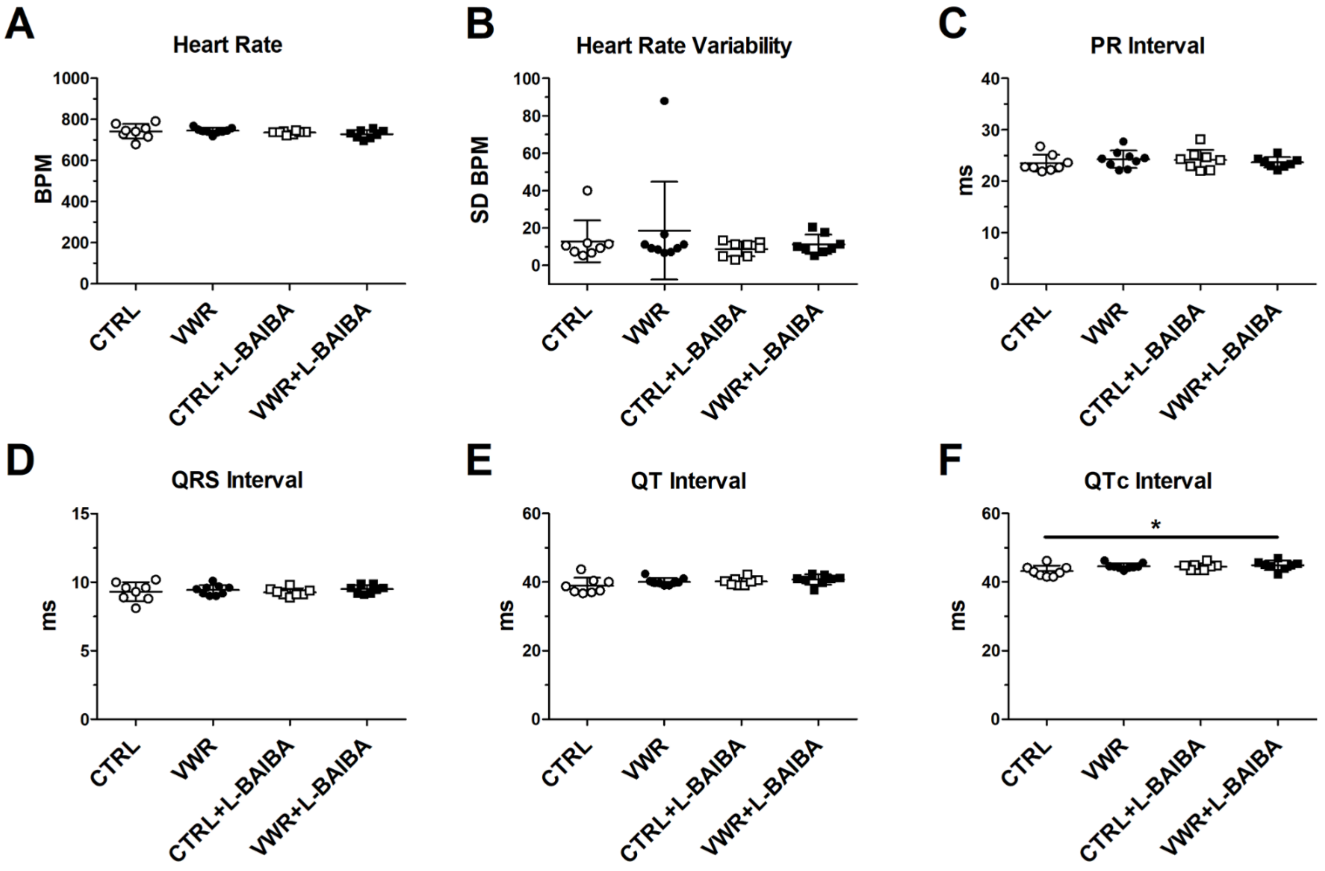

Supplementary Figure 1. Heart ECG properties after the three-month intervention period. Conscious ECG properties including (**A**) resting heart rate in beats per minute (BPM), (**B**) heart rate variability as measured by standard deviation of the heart rate (SD BPM), (**C**) PR interval, (**D**) QRS interval, (**E**) QT interval, and (**F**) QT interval corrected for heart rate (QTc interval). *p<0.05, One-way ANOVA. n = 7 CTRL, n = 8 VWR, n = 8 CTRL+L-BAIBA, n = 8 VWR+L-BAIBA. Mean ± SD.

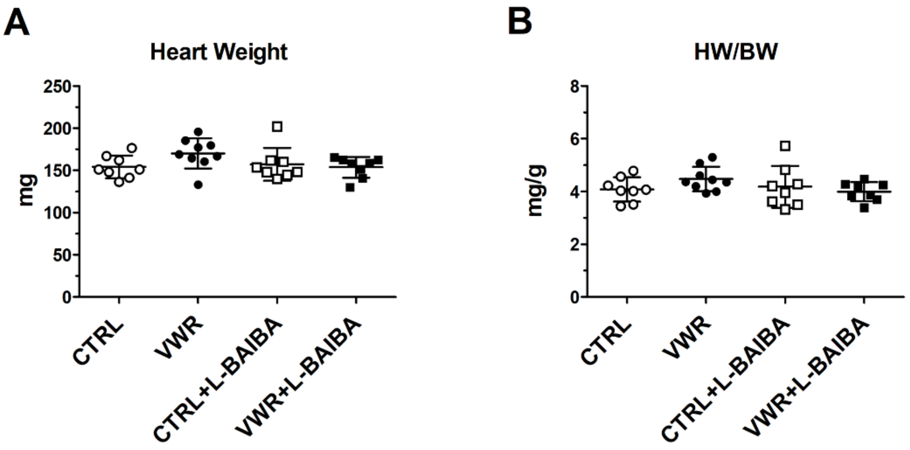

Supplementary Figure 2. Heart size after the three-month intervention period. (**A**) Average heart weight and (**B**) heart weight to body weight ratio (HW/BW). One-way ANOVA, $p>0.05$. n = 8 CTRL, n = 9 VWR, n = 8 CTRL+L-BAIBA, n = 8 VWR+L-BAIBA. Mean ± SD.

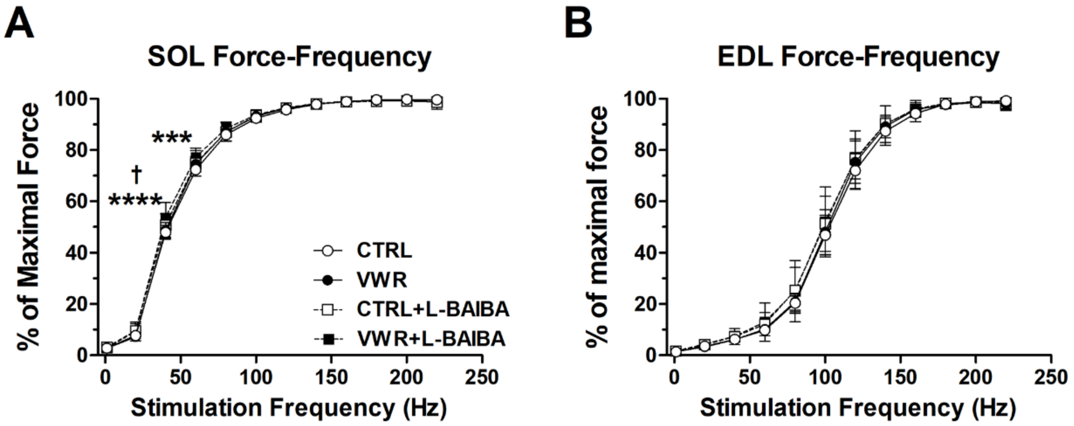

Supplementary Figure 3. *Ex vivo* muscle force-frequency after the three-month intervention period. Force-frequency relationships for soleus muscle (SOL) (**A**) and EDL muscle (**B**) expressed as a percentage of maximal force. ****$p<0.0001$, ***$p<0.01$ CTRL vs. VWR+L-BAIBA; †$p<0.001$ VWR vs. VWR+L-BAIBA, two-way ANOVA with Bonferroni. n = 8 CTRL, n = 9 VWR, n = 8 CTRL+L-BAIBA, n = 8 VWR+L-BAIBA. Mean ± SD.

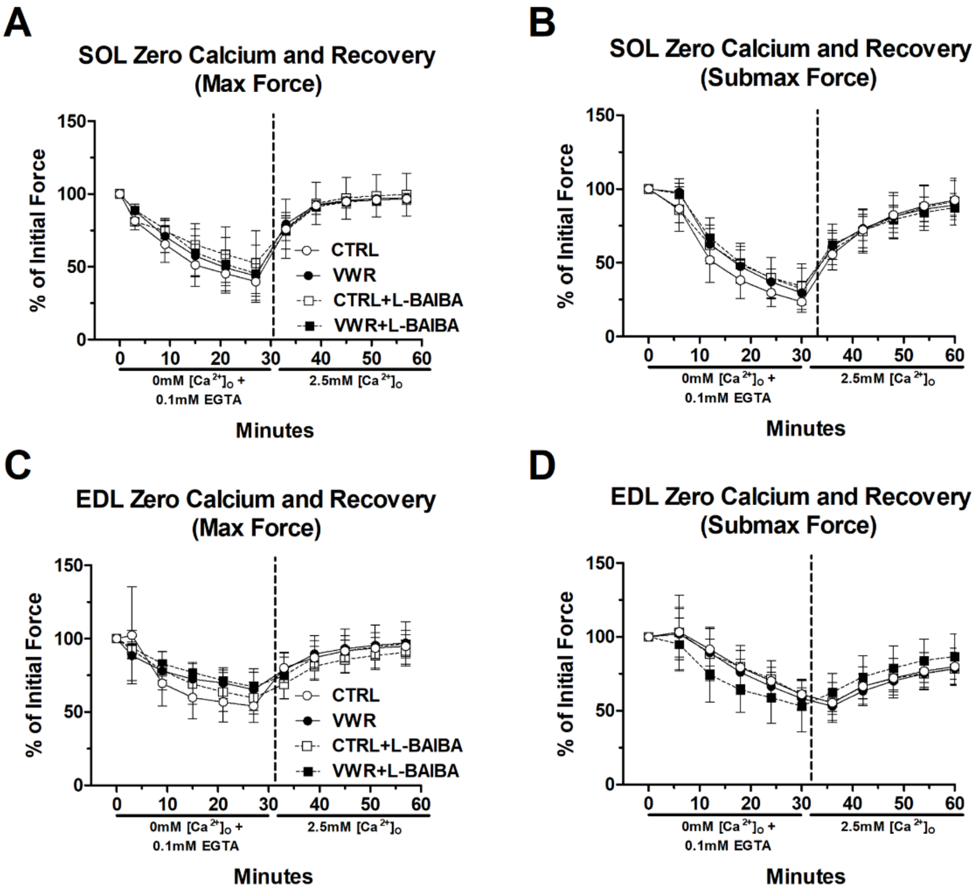

Supplementary Figure 4. Muscle dependence upon extracellular calcium for *ex vivo* contractility after the three-month intervention period. Contractile force production in buffer containing 0 mM Ca^{2+} and after restoration to normal Ca^{2+} levels in soleus (SOL) muscle at maximal force (**A**) and submaximal force (**B**), and in EDL muscle at maximal force (**C**) and submaximal force (**D**). Data is expressed as a percentage of initial force at baseline before 0 mM Ca^{2+}. Two-way ANOVA, $p>0.05$. n = 8 CTRL, n = 9 VWR, n = 8 CTRL+L-BAIBA, n = 8 VWR+L-BAIBA. Mean ± SD.

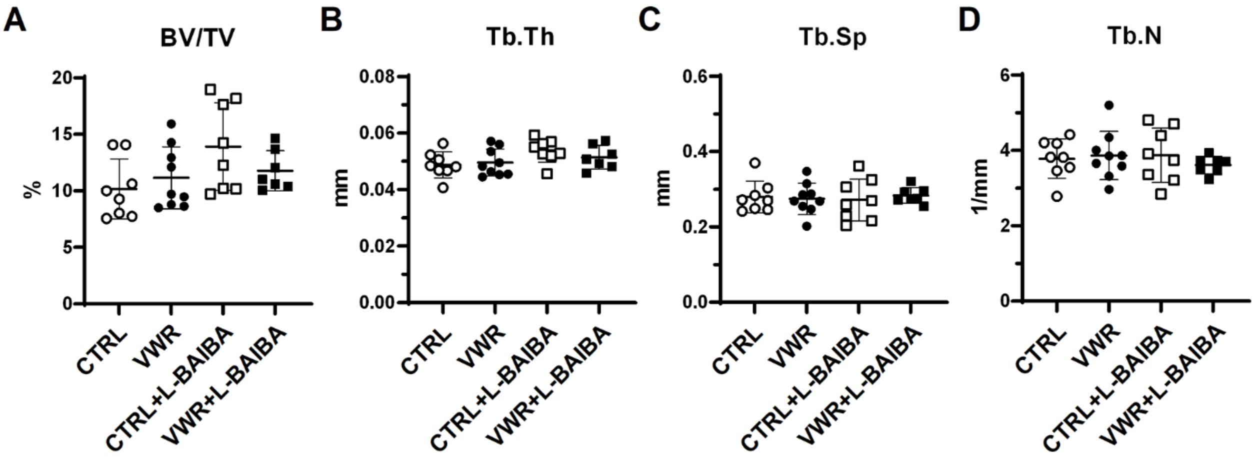

Supplementary Figure 5. Baseline µCT analysis of tibia trabecular bone parameters. (**A**) Trabecular bone volume fraction (BV/TV), (**B**) trabecular bone thickness, (**C**) trabecular bone separation, and (**D**) trabecular bone number, measured at baseline prior to the beginning of the study. One-way ANOVA, $p>0.05$. n = 8 CTRL, n = 9 VWR, n = 8 CTRL+L-BAIBA, n = 8 VWR+L-BAIBA. Mean ± SD.
